# *Phyllanthus* spp. Induces Selective Growth Inhibition of PC-3 and MeWo Human Cancer Cells through Modulation of Cell Cycle and Induction of Apoptosis

**DOI:** 10.1371/journal.pone.0012644

**Published:** 2010-09-08

**Authors:** Yin-Quan Tang, Indu Bala Jaganath, Shamala Devi Sekaran

**Affiliations:** 1 Department of Medical Microbiology, Faculty of Medicine, University of Malaya, Kuala Lumpur, Malaysia; 2 Biotechnology Centre, Malaysia Agricultural Research and Development Institute (MARDI), Serdang, Malaysia; University of Hyderabad, India

## Abstract

**Background:**

*Phyllanthus* is a traditional medicinal plant that has been used in the treatment of many diseases including hepatitis and diabetes. The main aim of the present work was to investigate the potential cytotoxic effects of aqueous and methanolic extracts of four *Phyllanthus* species (*P.amarus, P.niruri, P.urinaria* and *P.watsonii*) against skin melanoma and prostate cancer cells.

**Methodology/Principal Findings:**

*Phyllanthus* plant appears to possess cytotoxic properties with half-maximal inhibitory concentration (IC_50_) values of 150–300 µg/ml for aqueous extract and 50–150 µg/ml for methanolic extract that were determined using the MTS reduction assay. In comparison, the plant extracts did not show any significant cytotoxicity on normal human skin (CCD-1127Sk) and prostate (RWPE-1) cells. The extracts appeared to act by causing the formation of a clear “ladder” fragmentation of apoptotic DNA on agarose gel, displayed TUNEL-positive cells with an elevation of caspase-3 and -7 activities. The Lactate Dehydrogenase (LDH) level was lower than 15% in *Phyllanthus* treated-cancer cells. These indicate that *Phyllanthus* extracts have the ability to induce apoptosis with minimal necrotic effects. Furthermore, cell cycle analysis revealed that *Phyllanthus* induced a Go/G1-phase arrest on PC-3 cells and a S-phase arrest on MeWo cells and these were accompanied by accumulation of cells in the Sub-G1 (apoptosis) phase. The cytotoxic properties may be due to the presence of polyphenol compounds such as ellagitannins, gallotannins, flavonoids and phenolic acids found both in the water and methanol extract of the plants.

**Conclusions/Significance:**

*Phyllanthus* plant exerts its growth inhibition effect in a selective manner towards cancer cells through the modulation of cell cycle and induction of apoptosis via caspases activation in melanoma and prostate cancer cells. Hence, *Phyllanthus* may be sourced for the development of a potent apoptosis-inducing anticancer agent.

## Introduction

Cancer is a name given to group of diseases that arise from uncontrolled growth, spread of an abnormal cell and can result in death. It is extremely hard to treat due to several distinct classes of tumours that exhibit different responses to treatment and not all anticancer agents effectively give a positive response in every case [1]. Some have been reported to exhibit toxicity to normal cells, accompanied by undesirable effects such as vomiting, nausea and alopecia. Thus, ineffective anticancer agents have resulted in high death rates in cancer patients [2]. Melanoma is a type of skin cancer that arises from melanocytes, a pigment-producing tanning cell. Melanoma incidence and its mortality rate are high in fair-skinned populations in all parts of the world, including Australia, USA and UK [3]–[4]. Prostate cancer is the second leading cause of cancer deaths after lung cancer worldwide [3]. Currently, there are no effective treatments for both melanoma and prostate cancer, and as such intense research is required to obtain new anticancer agents for these cancers.

The high mortality in cancer patients has led many researchers to source for potential natural-product based therapeutic compounds [2]. Herbal plants and plant-derived medicines have been used as the source of potential anticancer agents in traditional cultures all over the world and are becoming increasingly popular in modern society [5]. The potential natural product-derived anticancer agents are known to possess various bioactive compounds such as roscovitine from red radish and flavopiridol from *Amoora rohituka*, a tropical tree that has shown tremendous effects in the treatment of cancers [6]–[8].

The plant of the genus *Phyllanthus* belongs to the family *Euphorbiaceae* and has been reported to have pharmacological effects such as antiviral activity against Hepatitis B and related hepatitis viruses [9]–[12], anti-bacterial activity [13], [14], anti-hepatotoxic or liver-protecting activity [15]–[19] as well as anti-tumour and anti-carcinogenic properties [16], [20]. In addition, it has also exhibited hypoglycaemia properties [21], [22]. Although the plant genus *Phyllanthus* has been shown to be beneficial for human health, but its effectiveness against cancer has not been fully elucidated.

One of the challenges in cancer treatment is that cancer possesses the ability to evade apoptosis (or programme cell death) which leads to its ineffectiveness as a cytotoxic drug to kill cancer cells. The apoptotic process is an important cell death mechanism in response to cytotoxic treatment and its induction is a highly desirable mode for an anticancer agent [23]. Cell cycle is a process that acts as a key to control growth and proliferation of a cell. The disruption of the cell cycle process will cause an imbalance between cell proliferation and cell death (apoptosis), subsequently leading to cancer development. Thus, cell cycle could serve as target for anticancer agent to halt uncontrolled proliferation of cancer cells and to initiate them to undergo apoptosis [24]. The cytotoxic effects of *Phyllanthus* extracts (aqueous and methanol) on growth inhibition against skin melanoma and prostate cancer cells in their cell cycle could partially explain their mode of activity. The objective of the present study was to determine the cytotoxic effect of *Phyllanthus* extracts on the proliferation of skin and prostate cancer cells and also to investigate the relationship of these antiproliferative effects with probable apoptosis and cell cycle modulation.

## Results

### Cytotoxic activity of aqueous and methanolic extracts of *Phyllanthus* species

In this study, we investigated the cytotoxic effects of crude aqueous and methanolic extracts of four different *Phyllanthus* species, *P.amarus, P.niruri, P,urinaria,* and *P.watsonii*, on two human cancers (MeWo and PC-3) as well as normal (CCD-1127Sk and RWPE-1) cell lines. The cytotoxic properties of the *Phyllanthus* extracts were determined using the MTS [3-(4,5-dimethylthiazol-2-yl]-5-(3-carboxymethoxyphenyl)-2-(4-sulfophenyl)-2H-tetrazoliuminner salt) reduction assay. The principle behind this assay is based on the reduction ability of a soluble tetrazolium salt, by mitochondrial dehydrogenase enzyme of viable cells, into a coloured soluble formazan product that can be measured spectrophotometrically. The half-maximal inhibitory concentration (IC_50_) value was determined from the constructed dose-response curve and set as a parameter for cytotoxicity.

Table 1 shows the comparison of IC_50_ values for both crude aqueous and methanolic extracts of the four *Phyllanthus* species on both human cancer (MeWo and PC-3) and normal (CCD-1127Sk and RWPE-1) cell lines. Result reveals the presence of cytotoxic effects of *Phyllanthus* species on both skin melanoma and prostate cancer cells, where the respective IC_50_ values of *Phyllanthus* extracts were determined. In comparison, the plant did not show any significant cytotoxic effects on normal human cells, while, the standard anticancer drugs (5′Fluorouracil and Doxorubicin) showed stronger cytotoxic effect than the *Phyllanthus* extracts, but exhibits toxicity on normal cells (Table 1). Among the four *Phyllanthus* plant species, *P.urinaria* showed strongest cytotoxic effect, followed by *P.watsonii, P.niruri* and *P.amarus*. In addition, the IC_50_ values of the methanol extracts of *Phyllanthus* species were noticeably lower than the aqueous extracts on both cancer cells indicating that the methanol extract is more cytotoxic than aqueous extract.

**Table 1:** IC_50_ values of *Phyllanthus* extracts and standard anticancer drugs on both human cancer and normal cell lines.

		IC_50±_ SEM (μg/ml)			
		Skin cells		Prostate cells	
*Phyllanthus* species	Extracts	Cancer (MeWo)	Normal (CCD-1127Sk)	Cancer (PC-3)	Normal (RWPE-1)
*P.amarus*	Aqueous	193.3±1.3	>500	178.3±2.8	>500
	Methanol	133.3±2.9	>500	84.3±1.1	>500
*P.niruri*	Aqueous	260.0±2.4	>500	155.0±1.2	>500
	Methanol	153.3±2.6	>500	117.7±2.1	>500
*P.urinaria*	Aqueous	193.3±1.1	>500	155.7±2.1	>500
	Methanol	56.21±3.2	>500	54.2±2.1	>500
*P.watsonii*	Aqueous	160.0±3.2	>500	156.7±2.4	>500
	Methanol	100.7±2.0	>500	100.5±1.2	>500
5′-Fluorouracil		2.3±0.5	0.8±0.5	1.0 ±0.3	1.0±0.5
Doxorubicin		2.5±0.5	1.0±0.2	2.5 ±0.5	1.0±0.1

### Identification of polyphenols in *Phyllanthus* species

Methanol and water-soluble extracts obtained from various species of *Phyllanthus* were subjected to analysis by HPLC (High-Performance Liquid Chromatography) coupled with photodiode array (PDA) and MS-MS detection allowing the identification of polyphenol compounds (Table 2). Twelve main compounds were identified on the basis of their retention times, UV spectra, and parent mass spectra and secondary fragmentation patterns. The compounds detected were gallic acid, galloylglucopyronside, digalloylglucopyronside, trigalloylglucopyronside, tetragalloylglucopyronoside, corilagen, geraniin, rutin, quercetin glucoside, quercetin diglucoside,quercetin rhamnoside, and caffeolquinic acid.

**Table 2:** Polyphenol compounds detected in *Phyllanthus species*.

Compound	Retention time in water extract (w) or methanol extract(m)	[M-H] m/z	MS-MS fragmentation	*Phyllanthus* species
Gallic acid	3.8 (w)	169	125,169	*P.amarus, P.niruri, P.urinaria, P.watsonii*
Galloylglucopyronoside	2.8 (w)	331	125,169,211,271	*P.amarus, P.niruri, P.urinaria, P.watsonii*
Digalloylglucopyronoside	15.0 (w)	483	125,169,211, 271, 313	*P.amarus, P.niruri, P.watsonii*
Trigalloylglucopyronoside	23 (w), 13 (m)	635	125,169,211, 271, 313,465	*P.urinaria*
Tetragalloylglucopyronoside	15 (m)	787	169,211, 313, 465	*P.urinaria*
Corilagen	18 (w)	633	301, 125, 169	*P.amarus, P.niruri, P.urinaria, P.watsonii*
Geraniin	22 (w), 12 (m)	951	301, 125, 169, 463	*P.amarus, P.niruri, P.urinaria, P.watsonii*
Rutin	26 (w)	609	301, 179,151	*P.amarus, P.niruri, P.urinaria, P.watsonii*
Quercetin glucoside	27 (w)	463	301, 179,151	*P.amarus, P.niruri, P.urinaria, P.watsonii*
Quercetin diglucoside	9 (m)	625	463, 301	*P.niruri*
Quercetin rhamnoside	30 (w)	447	301, 151	*P.urinaria, P.watsonii*
Caffeolquinic acid	23 (w)	353	191	*P.amarus, P.niruri, P.urinaria, P.watsonii*

### DNA fragmentation

**Figure 1:**
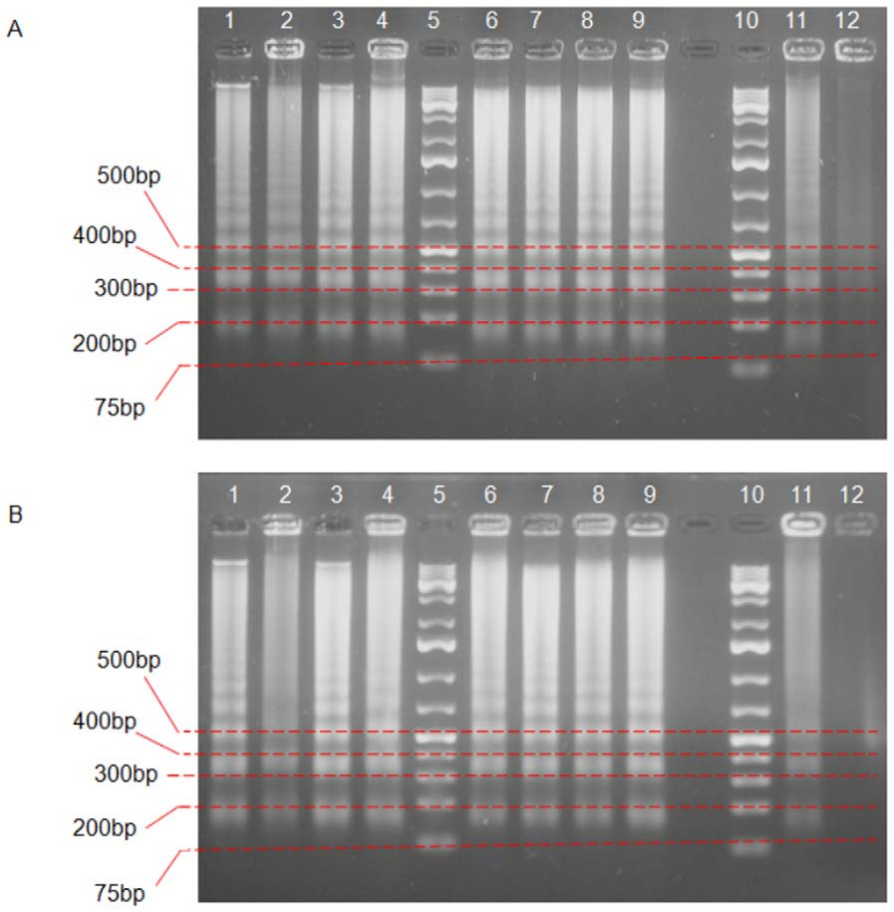
Apoptotic DNA fragmentation was observable in *Phyllanthus* extracts-treated MeWo and PC-3 cells. Lane 1 -4: aqueous and Lane 6 – 9: methanol extracts for *P.amarus, P.niruri, P.urinaria* and *P.watsonii*, in the order. Lane 5 and 10: 1 kb DNA marker, Lane 11: standard drug, where **A**) 5′Fluorouracil for MeWo and **B**) Doxorubicin for PC-3 cells. Lane 12: untreated cells.

One of the biochemical hallmarks in the apoptotic process is the formation of nuclear DNA fragmentation, which shows the presence of typical ladder DNA fragments of 180 – 200 base pairs and multiples thereof on an agarose gel. In contrast, random cleavage of DNA in necrotic cells will produce a diffuse smear upon electrophoresis of DNA. Hence, DNA gel electrophoresis method was used to determine the possible mode of cell death caused by *Phyllanthus* extracts. Figure 1A shows the presence of DNA fragments produced by treatment of MeWo cells with *Phyllanthus* extracts and a similar pattern was seen with the standard drug (5′Fluorouracil) as positive control. No ladder formation was observed in untreated cells. These phenomenons were also observed in PC-3 cell line for both extracts as shown in Figure 1B. Thus, this indicates that both extracts of *Phyllanthus* were capable of inducing apoptosis or programmed cell death on MeWo and PC-3 cells in response to the cytotoxic effects of *Phyllanthus*.

### TUNEL assay and Apoptotic Index

**Figure 2:**
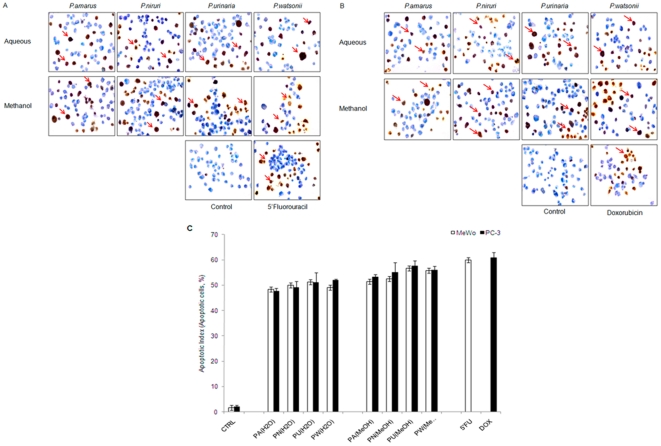
Induction of Apoptosis. TUNEL analysis of MeWo and PC-3 cancer cells after being treated with *Phyllanthus* extracts at 100× magnification. TUNEL-positive (apoptotic) cells were observable as brown stained cells (red arrow) in *Phyllanthus* extracts-treated **A**) MeWo and **B**) PC-3 cells and normal viable cells stain as blue colour. **C**) The graph shows the percentage of apoptotic index (%) of untreated and treated (*Phyllanthus* extracts and anticancer drugs) MeWo and PC-3 cancer cells from TUNEL analysis.

TUNEL (terminal deoxynucleotidyl transferase dUTP nick end labeling) assay is a technique to allow detection of apoptotic cells by labelling the free end of apoptotic DNA with a marker which can be visualized under light microscope. As shown in Figure 2, apoptotic cells were observed as brown-coloured cells in *Phyllanthus* extracts-treated MeWo (Figure 2A, arrowhead) and PC-3 (Figure 2B, arrowhead) cancer cells, their appearance were similar to apoptotic cells that were present in the positive controls, apoptotic-inducer anticancer drug (5′Fluorouracil and Doxorubicin), while viable cells were stained blue. This further confirms that *Phyllanthus* extracts were capable of inducing apoptosis on skin melanoma and prostate cancer cells. The populations of cell death can be calculated and expressed in a mathematical way, known as apoptotic index. From Figure 2C, the cell death percentage (apoptotic index) of treated-MeWo and PC-3 cells were markedly increased up to 50% compared to the control group at 72 hours of treatment with *Phyllanthus* extracts. In addition, the percentage of *Phyllanthus* extracts induced apoptotic cells was close to the anticancer drugs (5′Fluorouracil and Doxorubicin) with only 8% difference.

### *Phyllanthus* extracts-induced caspase-3/7 activations

**Figure 3:**
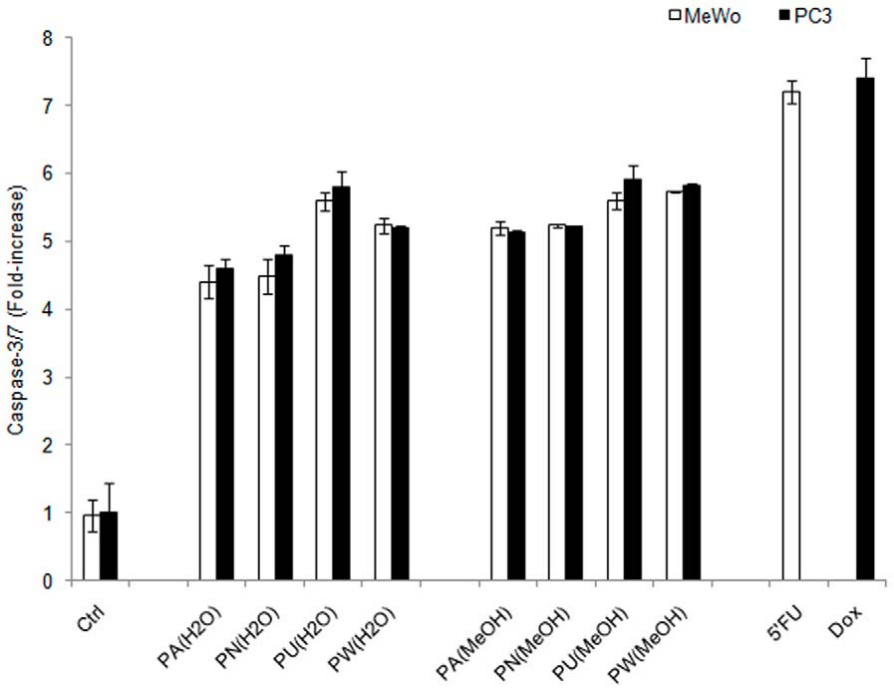
*Phyllanthus* extracts induce caspase-3 and -7 activation. The graph shows the levels of caspase-3 and -7 in treated and untreated groups of MeWo and PC-3 cells. **Bars show the mean ± SE**

An activation of caspases (aspartate specific cysteine protease) is one of the biochemical changes during apoptosis. The levels of caspase-3/7 induced by *Phyllanthus* treatment were markedly increased (3–4 folds) as compared to the untreated group (Figure 3) for both extracts of *Phyllanthus*. The level of caspase-3/7 of standard drugs, 5′Fluorouracil and Doxorubicin on MeWO and PC-3 cells, respectively, were 6-folds increase as compared to that of the control group and 0.5-fold higher than *Phyllanthus* extracts after 72 hours of treatment. These indicates that apoptosis induced by *Phyllanthus* extracts was mediated via caspases activation.

### *Phyllanthus* extracts-induced minimal necrotic effect

**Figure 4:**
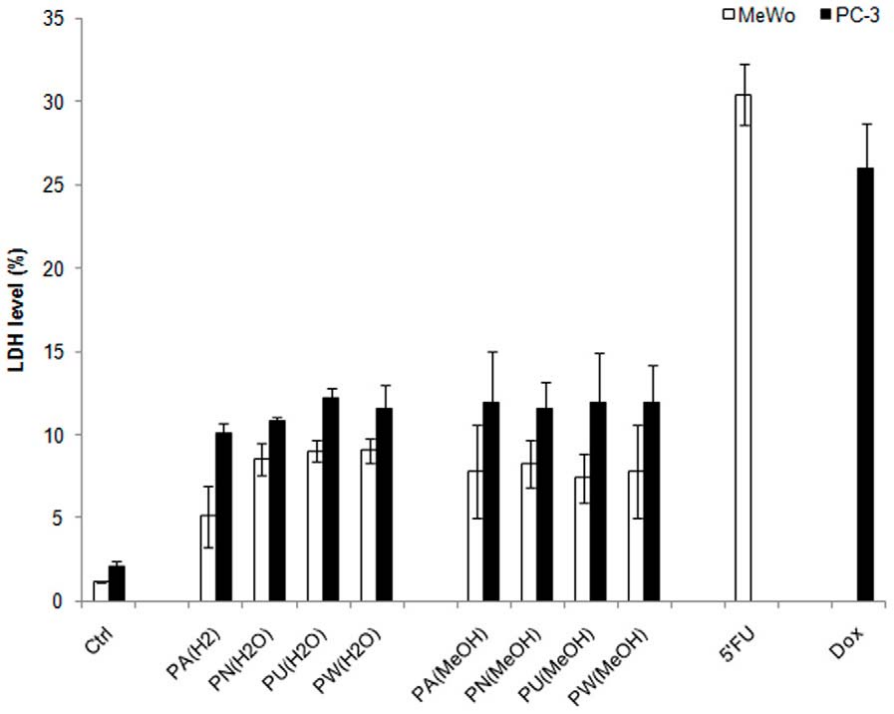
*Phyllanthus* extracts induce minimal necrotic effect. The graph shows the percentage of LDH levels in *Phyllanthus*-extracts treated MeWo and PC-3 cancer cells were higher than the untreated group after 72 hours of treatment. **Bars show the mean percentage ± SE**

Necrosis is another form of cell death that will provoke inflammatory response of surrounding cells through the leakage of intracellular contents. As shown in Figure 4, the percentage of LDH levels produced as a result of treatment with both extracts of *Phyllanthus* species was less than 10% for MeWo cells and less than 15% for PC-3 cells, as compared to that of the control groups after 72 hours of treatment. The necrotic effect of methanol extracts was more pronounced, where it was observed to be 4% higher than the aqueous extract of *Phyllanthus* species. This suggests that the *Phyllanthus* species possesses minimal necrotic effects and skin melanoma was less prone to display necrotic effect than prostate cancer cells. In contrast, the LDH level induced by the positive control (5′Fluorouracil and Doxorubicin) was 25% higher than the untreated cells and 18% in variation with the *Phyllanthus*–treated cells at 72 hours of treatment.

### *Phyllanthus* extracts induce cell cycle arrest followed by apoptosis

**Figure 5:**
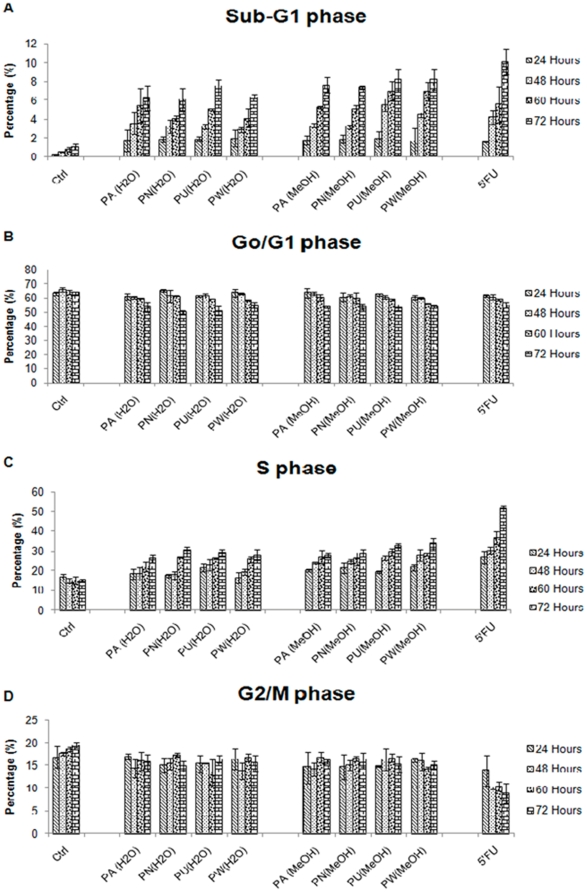
The kinetics of cell cycle distribution of *Phyllanthus* extracts treated MeWo cells. The percentage of *Phyllanthus* extracts-treated cells at **A**) Sub-G1, **B**) Go/G1, **C**) S, and **D**) G2/M phases of MeWo cells at different time intervals (24, 48, 60 and 72 hours) of treatment. **Bars show the mean percentage ± SE**

**Figure 6:**
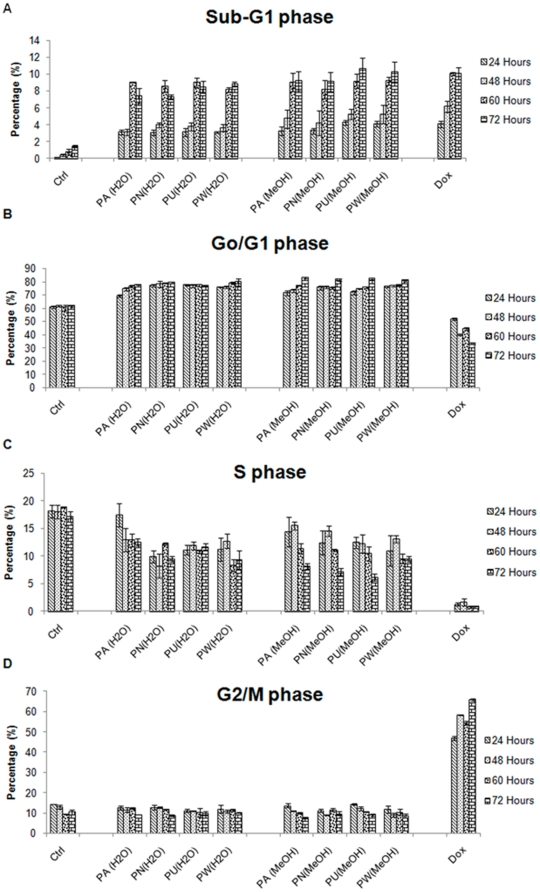
The kinetics of cell cycle distribution of *Phyllanthus* extracts treated PC-3 cells. The percentage of *Phyllanthus* extracts-treated cells at **A**) Sub-G1, **B**) Go/G1, **C**) S, and **D**) G2/M phases of PC-3 cells at different time intervals (24, 48, 60 and 72 hours) of treatment **Bars show the mean percentage ± SE**

To determine the phase of cell cycle that is inhibited by *Phyllanthus* plant extracts, both MeWo and PC-3 cells were treated at their respective IC_50_ values for 24, 48, 60 and 72 hours and then analysed by flow cytometry. The kinetics of the cell cycle distribution of treated and untreated groups of MeWo and PC-3 cells were shown in Figure 5 and 6, respectively. Changes in the distribution of treated cells in different phases of cell cycle were observable by 24 hours after being treated with *Phyllanthus* extracts for the both cancer cell lines.

As seen in the Figure 5, *Phyllanthus* extracts exhibited growth arrest at the S-phase in MeWo cells from 24 hours and remained evident after 72 hours of treatment and this was accompanied by an accumulation of cells in the Sub-G1 (apoptosis cells) phase for both aqueous and methanolic extracts. The percentage of apoptotic cells had increased in a time-dependent manner from 1.8% at 24 hours to 6.1% at 72 hours as compared to the control groups (Figure 5A). Meanwhile, the percentage of cells at the S-phase of treated MeWo cells was elevated to 15% above the controls at 72 hours of treatment (Figure 5C). Furthermore, the percentage of cells at Go/G1 and G2/M phases decreased with time upon treatment with *Phyllanthus* extracts due to the fact that treated cells have been arrested at S-phase and subsequently accumulated at Sub-G1 (apoptosis) phase (Figure 5B and 5D). However, the potency of *Phyllanthus* extracts to induce S-phase arrest was not as the strong as standard drug (5′Fluorouracil), with a 22.1% difference at 72 hours post treatment.

For PC-3 cells, *Phyllanthus* exhibited growth arrest at the Go/G1 phase at 72 hours post treatment with an accumulation of apoptotic cells in the Sub-G1 phase for both aqueous and methanolic extracts. The percentage of apoptotic cells increased from 3.4% at 24 hours up to 7.4% at 72 hours over the untreated groups (Figure 6A). The percentage of treated PC-3 cells at Go/G1-phase was 13.7% at 24 hours and this increased to 18.8% at 72 hours above the untreated groups (Figure 6B). However, the percentage of treated-PC-3 cells at the S and G2/M phases decreased with time of treatment (Figure 6C and 6D) due to the fact that treated PC-3 cells were arrested at Go/G1 phase and subsequently accumulated at Sub-G1 phases. While doxorubicin showed a G2/M phase arrest on PC-3 cells at 24 hours and remained evident after 72 hours of treatment.

## Discussion

Man has used plants extensively for treating various kinds of diseases since ancient times [7]. Herbal plants and plant-derived medicines have been widely used in traditional cultures all over the world and have gained popularity in modern society as natural alternatives to produce new potential therapeutic compounds for combating diseases [5]. Sixty percent of today's available anticancer drugs originated from natural products, and their derivatives (including antibiotics). This has resulted in greater confidence in natural products as important sources for the development of effective anticancer agents [7].

In the present study, both aqueous and methanol extracts of four plant species of *Phyllanthus* displayed cytotoxic effects on human skin melanoma (MeWo) and prostate (PC-3) cell lines. The variations in the IC_50_ values of *Phyllanthus* extracts against melanoma and prostate cancer cells might be due to the differing levels of bioactive compounds present in each *Phyllanthus* species such as quercetin, gallic acid, and caffeolquinic acid that have been proven to possess anticarcinogenic effects [16], [25]–[27]. In addition, the data obtained suggest that the methanolic extract of *Phyllanthus* seemed to have more pronounced cytotoxic effect than aqueous extract, indicating the methanol-soluble bioactive compounds contained in *Phyllanthus* are probably more potent than the aqueous-soluble bioactive compounds in killing cancer cells. Among the four *Phyllanthus* species used, *P. urinaria* showed the strongest cytotoxic effect. This could be associated to its exclusive content of trigalloylglucopyronoside and tetragalloylglucopyronosid. Interestingly, *Phyllanthus* extracts did not cause any significant changes on the cell viability of both normal human skin (CCD-1127Sk) and prostate (RWPE-1) cell lines. These findings correlate with studies carried out by Huang et al. in which *Phyllanthus* plants displayed selective killing against cancer cells [28]. This selective cytotoxic effect is an important criterion because the currently available drugs target normal cells as well and leads to side effects.

The life and death of a cell is controlled by the cell cycle, which is tightly regulated by cyclin, cyclin-dependent kinases (CDK), CDK inhibitors (CDKI) and other tumour suppressor genes. Thus, deregulation of cell cycle will lead to abnormal proliferation of DNA-damaged cells and evasiveness of apoptosis [29]. *Phyllanthus* extracts disrupted the cell cycle of MeWo cells at S-phase, thereby implying that they might have interfered with DNA synthesis, halting the progression of cell cycle at S-phase and leading to apoptosis. The S-phase arrest by *Phyllanthus* extracts on MeWo cell could be due to: (1) inhibition of cdc25 enzymes [16], [30]–[32], or (2) inhibition of DNA Topoisomerase II which leads to activation of caspases [16], [33]. Thus, high levels of caspase-3 and -7 activities were detected after 72 hours post treatment of *Phyllanthus* extracts. However, S-phase arrest exhibited by *Phyllanthus* extracts were not as pronounced as 5′Fluorouracil, which is an S-phase sensitive skin anticancer drug, indicating that *Phyllanthus* extracts probably kill melanoma cells in other ways besides disrupting the cell cycle, such as alterations in melanoma's cell signalling pathways including FAS pathway [34]. Further investigations will address the mode of actions of *Phyllanthus* extracts on melanoma cells.

*Phyllanthus* extracts exerted their growth arrest on treated-PC-3 cells by accumulating the cells at Go/G1-phase, implying that *Phyllanthus* extracts may interfere with protein synthesis of PC-3 cells thus halting their progression from G1 to S phase during their cell cycle and subsequently initiating apoptosis. This could be due to the inhibitory effects on MDM2 protein, which reduces cell proliferation and induces apoptosis by the elevation of p21, Bax and pRb levels as well as reduction of hyperphosphorylated Rb and E2F1 [35]. *Phyllanthus*-induced apoptosis in PC-3 cells might be associated with elevations of Bax and Fas receptor/ligand gene expression as proposed by Huang et al. [25]. The elevations of Bax protein is associated with the involvement of mitochondrial (intrinsic) pathway in apoptosis which involves the reduction of mitochondrial membrane potential, cytochrome C releases and subsequently leading to caspases activation [36]. High levels of caspase-3 and -7 activities were detected in cells treated with *Phyllanthus* extracts. The Go/G1-phase arrest on PC-3 by *Phyllanthus* extracts might be associated to its high content of polyphenol compounds. The 12 polyphenols identified in *Phyllanthus*, are classified into four main groups, namely, ellagitannins, gallotannins, flavonoids and phenolic acids with ellagitannins being the most abundant group of compound. Ellagitannins are also abundantly found in pomegranate and have been studied quite thoroughly for its varied therapeutic effects including its cancer suppressing properties [37]. Geraniin, the main ellagitannin found in all the P*hyllanthus* species, have been shown to contribute to growth arrests on other cancers including colon cancer [38], [39]. However, the standard drug for prostate cancer, Doxorubicin, disrupted the growth of cancer cells by arresting them at G2/M phase and eventually initiating apoptosis by a variety of mechanisms such as inhibition on topoisomerase enzymes as seen in other studies [40], [41].

The activation of caspase-3 and -7 activities in treated cancer cells during apoptosis results in: (1) inactivation of enzyme poly (ADP-ribose) polymerase or PARP, and (2) activation of caspase activated DNase (CAD), subsequently causing DNA fragmentation, which is one of characteristics of apoptosis [42], [43]. Thus, elevation of these caspases after *Phyllanthus* treatments allows the appearance of apoptotic DNA fragments on agarose gel which was further confirmed with the presence of TUNEL-positive cells. Hence, the cytotoxic effects of *Phyllanthus* extracts on human skin (MeWo) and prostate (PC-3) cancer cell lines was mediated via an apoptotic mechanism with activation of caspase-3 and -7 and this could be due to the presence of gallic acid found in *Phyllanthus* extracts. Gallic acid has previously been proven to induce apoptosis via caspase-3 activation [44].

In natural product-based drug discovery and development, necrosis may occur along with apoptosis. This has been shown in many anticancer drugs such as cladribine, cisplatin, doxorubicin and 5′fluorouracil, which possess both apoptotic and necrotic effects [45], [46]. The LDH are a group of enzymes that are present in our body and their abnormally high level indicates health problems. In cells, LDH are mainly produced by the mitochondria and play an important role in oxidation of lactate while reducing pyruvate [47]. In necrotic cell death, plasma membrane integrity is lost and this leads to the leakage of cytoplasmic contents into the extracellular environment causing an inflammatory reaction [23]. Measurements of LDH enzyme as an indicator of necrosis, demonstrated that *Phyllanthus* besides having apoptotic activity, also possess minimal capacity of inducing necrotic cell death on both MeWo and PC-3 cells. Taken together, the results indicate that *Phyllanthus* plant possesses dual-capability of cell death, perhaps due to the crude nature of the *Phyllanthus* extracts, where all potential bioactive compounds are mixed together and may act individually or in synergism, thus contributing to this dual-capability of cell death' effects.

These bioactive compounds are believed to possess cytotoxicity towards cancer cells through its ability to disrupt the cell growth of cancer and initiate them to undergo apoptotic cell death. Although the detailed or underlying mechanisms of selectivity of *Phyllanthus* plant against skin and prostate cancer cells is still unclear, our findings have revealed that *Phyllanthus* plant exerts its growth inhibition towards cancer cells through modulation of cell cycle and induction of apoptosis via caspases activation. Further purifications of *Phyllanthus* extracts and investigations of the apoptosis pathway are needed to reveal the exact mode of action of *Phyllanthus* plant for its anti-cancer properties.

## Materials and Methods

### Plant Extracts and Standard Drugs

Aqueous and methanolic extracts of four *Phyllanthus* species (*P.amarus, P.niruri, P.urinaria, and P.watsonii*) were provided by Dr. Indu Bala, Biotechnology Centre, MARDI. Freshly harvested plant samples were washed, dried in room temperature and then freeze dried. For the aqueous extract, dried plant sample were soaked with ultra pure water, while absolute methanol was used for the methanolic extract preparation. The samples were then homogenized with extraction buffer and the supernatant collected after three rounds of extraction. Doxorubicin and 5′Fluorouracil were the standard drugs used as positive controls in this study. Both tested samples; plant extracts and standard drugs were stored at −20°C.

### High performance liquid chromatography coupled with electronspray ionization (ESI) and mass spectrometry (LC-MS- MS) analysis

For water extracted samples, 2 ml of supernatant was dried in a vacuum concentrator (Concentrator 5301 eppendorf, Germany) and re-dissolved into 20 mg/ml with 30% methanol before being subjected for LC-MS-MS analysis. For those samples extracted with methanol, total supernatant was evaporated using rotary evaporator (Rotavapor RII, BUCHI, Switzerland) and re-dissolved again with 20% methanol. Samples then were separated with solid phase extraction (SPE) column (LiChrolut RP-18 1000 mg/6 ml, Merck Germany) with mobile phase of 60% methanol and 70% methanol. All elutes were concentrated to 0.5 ml, then diluted 8 times with 40% methanol before being subjected for LC-MS-MS analysis.

Samples were separated using HPLC system comprising of a HPLC binary pump, an autosampler injector compartment and diode array detector (DAD) (1200 series, Agilent Technologies, Germany). Separations were carried out using a reverse phase C-18, 150 mm ×4.6 mm i.d, 5 µm particle size Thermo Hypersil GOLD column (Thermo Scientific, UK). Separation was developed using a mobile phase of 0.1% formic acid in water (solvent A) and 0.1% formic acid in acetonitrile (solvent B) with a gradient setting of solvent B: 5% (5 min), 5–90% (60 min), 5% (4 min) at flow rate of 1 ml/min. The injection volume was set at 20 µl and the detections were both at 280 nm and 360 nm. For mass spectrometry analysis, 3200 QTrap LC/MS/MS system (Appiled Bioscience – MDS Sciex) was used with the iron source and voltage was maintained at 500°C and −4.5 kV for negative ionization, respectively. Nitrogen generator was set to be operated at 60 psi curtain gas flow, 90 psi source gas flow and 60 psi exhaust gas flow. Two types of scanning modes were chosen: enhance mass spectrometer (EMS) and enhance ion product (EPI) for a full scan mass spectra ranging from *m/z* 100–1200.

### Cell culture

Skin melanoma MeWo (HTB-65) cell, prostate cancer PC-3 (CRL-1435) cells, normal human skin CCD-1127Sk (CRL-2565) and prostate RWPE-1 (CRL-11609) cell lines were purchased from American Type Culture Collection (ATCC). The four cell lines were cultured with different media, EMEM (Eagle's minimum essential medium) for MeWo cells, RPMI-1640 (Roswell Park Memorial Institute) for PC-3 cells, and keratinocyte growth medium, CC-4455 (Lonza, USA) for RWPE-1 cells and DMEM (Dulbecco's modified Eagle Medium) for CCD-1127Sk cells. Growth media was supplemented with 10% heat-inactivated foetal bovine serum (FBS, Gibco). Cells were maintained in humidified air with 5% CO_2_ at 37°C. Cells were harvested using 0.25% trypsin (Hyclone) when they reach 70–80% confluency in culture flasks. Cells undergoing exponential growth were used throughout the experiments.

### Cytotoxicity screening

Cells were seeded at optimal cell density into sterile 96-well plates and incubated overnight for attachment. The stock solution of *Phyllanthus* extracts was serially diluted and added to the cells to reach a final concentration at a range of 15.63–500 µg/mL and finally incubated for 72 hours. Anticancer drugs, Doxorubicin (Calbiochem) and 5′Fluorouracil (Duchefa) were used as positive controls for PC-3 and MeWo cells, respectively. After 72 hours, the cytotoxicity screening was performed using CellTiter 96® AQ_ueous_ Non-Radioactive Cell Proliferation Assay kit (Promega, USA) according to the manufacturer's instructions. Briefly, MTS/PMS solution was added in equal volumes and the plate was stored in the dark for 1 hour before determining their absorbance at 490 nm with the reference wavelength at 600 nm by using GloMax Multi Detection System (Promega, USA). The absorbance value that was determined for untreated cells was based on 100% viable cells. Each measurement was performed in triplicates. The IC_50_ values of *Phyllanthus* extracts on both the cancer cell lines were determined and used in subsequent experiments.

### Apoptosis analysis

#### DNA fragmentation

*Phyllanthus* extracts treated-cancer cells were lysed with lysis buffer (1M Tris-HCI, 0.5M EDTA, Triton-X and distilled water) and incubated at 4°C for 35 minutes. The DNA sample was extracted by mixture of phenol:choloroform:isoamyl (25∶24∶1) and precipitated with equal volume of ice-cold isopropanol. Cells were then centrifuged for 30 minutes at 10, 000 rpm and pellet was resuspended with deionized water-RNase solution (10 mg/ml RNase I). The DNA sample was incubated at 37°C for 30 minutes and electrophoresed on a 1.2% agarose gel containing 1 µg/ml ethidium bromide (Sigma) for 1 hour at 100 volts. Finally, the apoptotic DNA fragments were visualized under a UV transilluminator (Vilbir Lourmat) and photographed (Olympus).

### TUNEL assay and Apoptotic Index (AI)

The presence of apoptotic cells were determined using the ApopTag Plus Peroxidase *In Situ* Apoptosis Detection Kit (Chemicon International), which is based on the principle of TUNEL. Briefly, trypsin-detached cells were fixed with 1% paraformaldehyde in PBS (pH 7.4) and dried on a silanized glass slide. Pre-cooled ethanol:acetic acid (2∶1. v/v) was used for post-fixation of cells on the slide for 5 minutes at −20°C. Endogenous peroxidase was quenched by 3% H_2_O_2_ added at room temperature for 15 minutes. The apoptotic DNA fragments were labelled with digoxigenin antibody with TdT (terminal deoxynucleotidyl transferase) enzyme and then conjugated with anti-digoxigenin antibody. The labelled peroxidase-apoptotic DNA fragments were then treated with peroxidase substrate, diaminobenzidine (DAB) to produce a permanent, localized brown-coloured stain. Methyl green was used to counterstain the cells to further differentiate the apoptotic cells from viable normal cells. The slide was observed under light microscope and photographed at a magnification at 100X. The number of apoptotic cells were calculated from a total of at least 1000 apoptotic cells at 100× magnification and presented as an apoptotic index. Apoptotic cells were identified in TUNEL assay by apoptosis characteristic morphological changes such as cell shrinkage, membrane blebbing, and chromatic condensation. The percentage of apoptotic index was presented as the mean of experiments conducted in triplicates.

### Caspase-3 and -7 detection

Caspase-3/7 activity of treated-cells was detected by Caspase-Glo®-3/7 Assay system kit (Promega, USA) after 72 hours of treatment with *Phyllanthus* extracts. Briefly, an equal volume of Caspase-Glo® 3/7 reagent was added and luminescence signal was recorded using GloMax Multi Detection System (Promega, USA) after incubation at room temperature for 1 hour. The percentage of caspase-3/7 of *Phyllanthus* extract-treated cell was determined, where the background luminescence associated with the culture media and assay reagent (blank reaction) was subtracted from the experimental values of 96-well white walled plate. The percentage activity of caspase-3/7 level was presented as the mean of experiments conducted in triplicates.

### LDH measurements

LDH enzyme was used as an indicator for necrosis and was measured using a CytoTox-One (TM) Homogenous Membrane Integrity kit (Promega, USA) according to the manufacturer's instruction. Briefly, an equal volume of CytoTox reagent was added and incubated at room temperature for 10 minutes. Fifty microlitres of stop solution was added and fluorescence signal was measured at an excitation wavelength of 560 nm and an emission wavelength of 640 nm by using GloMax Multi Detection System (Promega, USA). The LDH levels were presented as the mean of experiments conducted in triplicate manner.

### Cell cycle analysis

Cells treated with respective IC_50_ value of *Phyllanthus* extracts were incubated at different time intervals; 24, 48, 60 and 72 hours. The floating and trypsin-detached cells were collected and fixed in 70% ethanol. The ethanol-fixed cells were spun down and washed twice with ice-cold PBS. The cell pellet was stained with propidium iodide (10 µg/ml) (Sigma) in PBS (Flowlab) containing RNase A (100 µg/ml) and was incubated in a 37°C water bath (Memmert) for 30 minutes in the dark before being analyzed on a FASCalibur flow cytometer (Becton Dickinson, USA). The percentage of cells at the different phases of the cell cycle was quantitated using WinMDI software. The percentage of hypodiploid cells (Sub-G1) over total cells was calculated and expressed as percentage of apoptotic cells.

### Data analysis

Results were expressed as the mean ± standard error (SE) of data obtained from triplicate experiments.
